# Lumbar Stenosis Spinal Surgery-Associated Cerebrospinal Fluid Leak Without Headache: An Autobiographical Case Report

**DOI:** 10.7759/cureus.25253

**Published:** 2022-05-23

**Authors:** Philip R Cohen, Stephen M Dorros

**Affiliations:** 1 Dermatology, University of California, Davis Medical Center, Sacramento, USA; 2 Radiology, University of California San Diego, La Jolla, USA

**Keywords:** spinal surgery, seroma, lumbar stenosis, leak, headache, duro-cutaneous fistula, dural tear, cutaneous, cerebrospinal fluid, autobiographical case report

## Abstract

Lumbar spinal stenosis, a narrowing of the spinal canal around the spinal neurovascular structures, is a common etiology for lower back and leg pain in older people. Sciatica, a frequent symptom of lumbar spinal stenosis, typically presents with sharp and/or aching pain that originates in the buttock, extends to the thigh, and radiates into the foot and toes; in addition, it can be accompanied by weakness of the associated lower extremity. In individuals with sciatica-related persistent symptoms or functional limitations or both, spinal decompression surgery may be necessary. A cerebrospinal fluid leak is a potential complication of lumbar spinal stenosis surgery; it is frequently--yet not always--accompanied by a postural headache. The cerebrospinal fluid leak can result from an intraoperative tear or postoperatively. Albeit a more common adverse event after body contouring surgery, seroma--a postoperative serous fluid collection that is usually detectable as a palpable or visible fluid wave on clinical examination--has also been observed as a complication following lumbar spinal stenosis surgery. A man who experienced an intra-operative accidental dural tear during lumbar spinal stenosis surgery is described. A large cerebrospinal fluid leak that involved both the laminectomy bed and the subcutaneous tissue of his back subsequently developed; the leak eventually presented as duro-cutaneous fistulas without headache. His doctors misinterpreted the cerebrospinal fluid leak as a seroma; this may have occurred since not only did the color of the persistent and continuously dripping fluid varied from being clear to slightly tinged pink, but also the patient never had a headache or any other symptoms associated with a cerebrospinal fluid leak. When his lower back was appropriately evaluated with magnetic resonance imaging, the diagnosis of a large cerebrospinal fluid leak was established. In conclusion, lumbar spinal stenosis back surgery can be associated with postoperative complications, including cerebrospinal fluid leak and--less frequently--seroma. However, following lumbar spinal stenosis surgery, the absence of a headache does not exclude the possibility of a cerebrospinal fluid leak. Also, the presence of fluid leaking from the surgical site after lumbar spinal stenosis back surgery should not only prompt the clinician to entertain the possibility of a surgery-associated cerebrospinal fluid leak but also to obtain additional diagnostic studies--such as magnetic resonance imaging--to establish the diagnosis.

## Introduction

Lumbar spinal stenosis is a narrowing of the spinal canal around the spinal neurovascular structures. It has a prevalence of approximately 11 percent in United States adults and commonly presents with sciatica symptoms. Management ranges from conservative non-surgical modalities to operative intervention [1-8].

A cerebrospinal fluid leak is a potential complication following back surgery; a dural tear during the procedure may place the patient at greater risk for this post-operative adverse event. Headache is a common symptom that typically accompanies a cerebrospinal fluid leak. However, not all patients with a cerebrospinal fluid leak have an associated headache [9-20].

A man with bilateral and severe lumbar stenosis affecting multiple spinal disc levels had back surgery during which a dural tear experienced during the procedure was repaired; he never experienced any postoperative headache. However, his cerebrospinal fluid leak clinically presented on a postoperative day seven as continuous draining fluid onto the skin of his back within the area of his prior operative wound. Hence, the absence of a headache does not exclude the possibility of a cerebrospinal fluid leak--especially in the setting of an intraoperative tear of the dura and fluid draining onto the skin at the surgical site.

## Case presentation

The patient, a 58-year-old man, presented to the emergency center for an evaluation regarding the fluid that slowly yet continuously was seeping from the suture sites on his lower back. He was afebrile, and his vital signs (including blood pressure, pulse, and respirations) were normal. He had not experienced a headache during the prior 12 days after his back surgery and currently did not have a headache.

**Figure 1:**
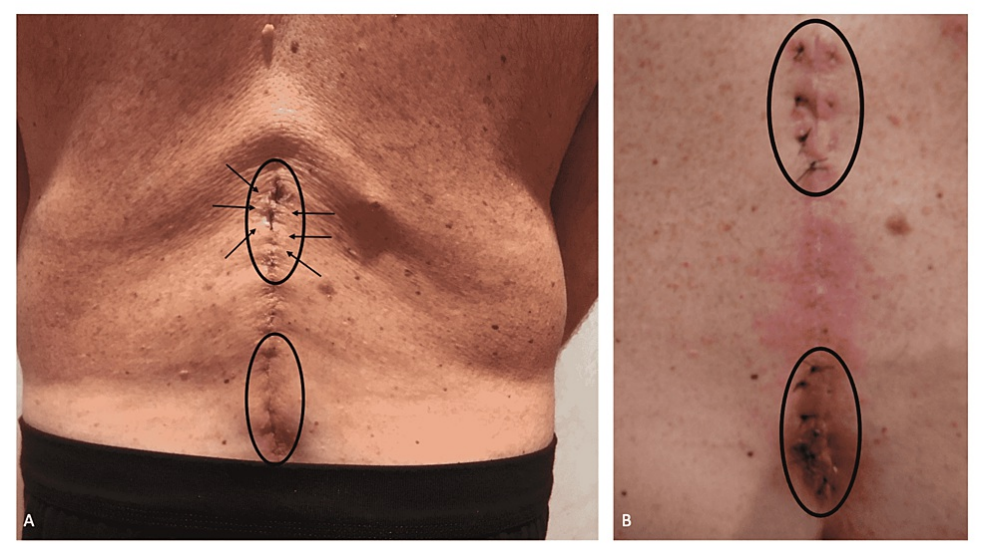
Clinical presentation of the cerebrospinal fluid leak draining onto the back skin and suture repair of the duro-cutaneous fistulas The lower back of a 58-year-old man, who had back surgery 12 days earlier, shows the sites where cerebrospinal fluid has leaked onto the skin (black ovals); superiorly (upper black oval), flakes of dried cerebrospinal fluid (black arrows) can be observed (A). Subsequently, sutures were placed deeply into the subcutaneous tissue to compress the area affected by the duro-cutaneous fistulas and thereby prevent any further leakage of cerebrospinal fluid to the skin’s surface (B).

Cutaneous examination of his lower back demonstrated two areas in which fluid was present. The fluid at the superior location had dried onto the skin surface. Clear fluid was sparsely present on the erythematous lower site (Figure 1A).

**Figure 2:**
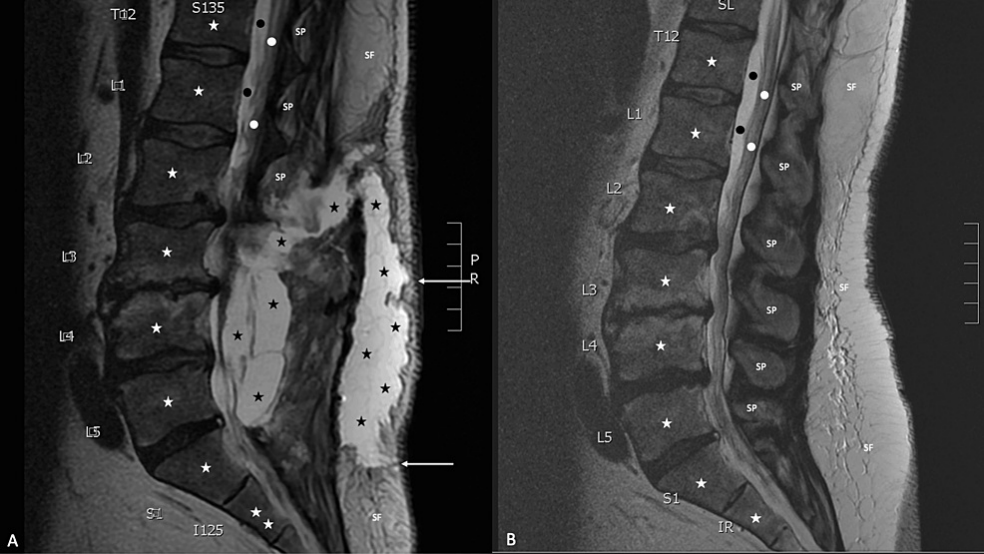
Post-surgical and pre-surgical sagittal views of magnetic resonance imaging with contrast of the lower back A magnetic resonance imaging of the lumber back of a 58-year-old man was performed on a 1.5 Tesla scanner, with sagittal short T1 inversion recovery, T1-weighted, and T2-weighted imaging 12 days after surgery to evaluated him for a post-operative cerebrospinal fluid leak (A). The sagittal view demonstrates the vertebral bodies (white stars) and spinal process (labeled SP) of the lower thoracic (labeled T12), lumbar (labeled L1 to L5) and upper sacral (labeled S1) vertebrae. The lower portion of the spinal cord (white dots) and cerebrospinal fluid in the dura (black dots) can also be seen. In addition, subcutaneous fat (labeled SF) can be noted. There are two large, connected collections of cerebrospinal fluid (black stars) in the soft tissue of the back; two tracts containing cerebrospinal fluid extend from the soft tissue collection to the skin surface (white arrows). The sagittal view of a similarly performed magnetic resonance imaging, from 18 months earlier, does not show any leakage of cerebrospinal fluid (B).

**Figure 3:**
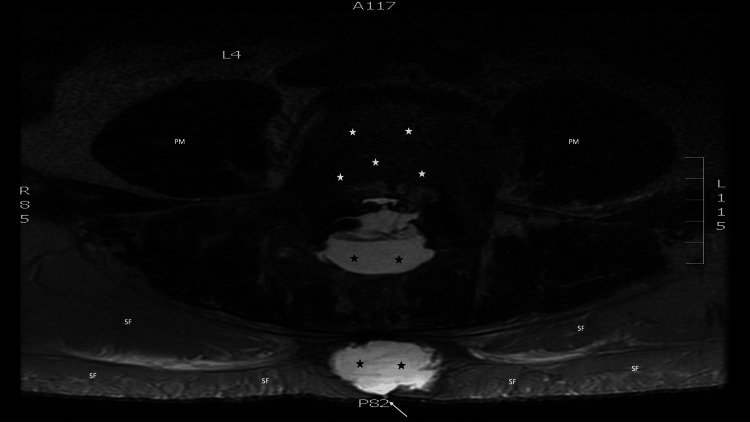
Axial view of magnetic resonance imaging showing the cerebrospinal fluid leak Twelve days after surgery, a magnetic resonance imaging of the lumber back of a 58-year-old man was performed on a 1.5 Tesla scanner, with axial T1-weighted and T2-weighted imaging. The axial view, at the level of the fourth lumbar vertebrae, demonstrates the vertebral body (white stars) and bilateral psoas muscles (labeled PM). Subcutaneous fat (labeled SF) can be seen. In addition, two collections of cerebrospinal fluid (black stars) are present in the soft tissue of the back; a tract (white arrow), extends from one of the soft tissue collections of cerebrospinal fluid to the surface of the skin surface.

Magnetic resonance imaging of the lumbar spine was performed--without and with ten milliliters of Gadavist intravenous contrast--on a 1.5 Tesla scanner, with not only sagittal short T1 inversion recovery, T1-weighted, and T2-weighted imaging (Figure 2A), but also axial T1-weighted and T2-weighted imaging (Figure 3). There was a large irregular fluid collection centered within the lumbar 3 to lumbar 5 laminectomy beds, with extension superiorly into the posterior paraspinal space surrounding the residual lumbar 2 processes, measuring 13 centimeters craniocaudal by three centimeters anteroposterior by 3.1 centimeters transverse; the mass effect of the fluid collection on the thecal sac resulted in bilateral compression of the cauda equina nerve roots and severe spinal canal stenosis at each disk level from lumbar 2 to lumbar 5. The first fluid collection communicates with a second large irregular fluid collection within the subcutaneous soft tissue, extending from lumbar 2 to sacral 1, measuring 14 centimeters craniocaudal by three centimeters anteroposterior by four centimeters transverse. In addition, focal dural discontinuity dorsally at the lumbar 4 to lumbar 5 levels was observed; also, edema and enhancement within the paraspinal musculature--attributed to the recent surgery--were present. 

In summary, the large cerebrospinal fluid collection located deep in the laminectomy bed communicated with another large collection of cerebrospinal fluid located more superficially in the subcutaneous tissue of the patient’s back; also, there was discontinuity of the dura. Correlation of the clinical history and magnetic resonance imaging findings established the diagnosis of a dural tear with a subsequent large cerebrospinal fluid leak. In addition, there was bilateral severe spinal canal stenosis at multiple disc levels with compression of the cauda equina nerve roots.

The magnetic resonance imaging changes of his cerebrospinal fluid leak were readily apparent when compared to his initial magnetic resonance imaging, performed 18 months earlier when he presented with bilateral pain that originated in his thighs and extended distally towards his feet (Figure 2B); the magnetic resonance imaging showed bilateral severe spinal canal stenosis at the following disc levels: lumbar 2 to lumbar 3, lumbar 3 to lumbar 4, and lumbar 4 to lumbar 5. In addition, there was mild spinal canal stenosis at the right lumbar 5 to sacral 1 level. He was referred to a senior, university-based orthopedic surgeon who specialized in back surgery for the management of his bilateral sciatica symptoms. After the initial consultation, a conservative approach to management was elected.

A computed tomography-guided epidural corticosteroid injection localized to the site of the most severely affected nerves was performed. All symptoms resolved; the patient was able to continue running. However, nearly a year later, symptoms recurred, and right leg weakness had developed; the patient was no longer able to stand up on the toes of his right foot. Another computed tomography-guided epidural corticosteroid injection resulted in symptom relief; yet, within three months, left leg weakness--similar to that of his right leg--developed. He met with the surgeon and decided to have surgery.

He had spinal surgery (consisting of decompression laminectomy without fusion) to treat the severe bilateral spinal stenosis that involved multiple vertebrae 12 days before his visit to the emergency center. During the operation, a surgical tear of the dura was repaired; he was restricted to the hospital bed and only allowed to lie on his back--with his feet slightly elevated--until the third post-operative day. He was able to urinate, defecate, and ambulate by the fifth postoperative day; he was discharged with a simple bandage covering the surgical site that was to remain in place for the next three days.

The area covered by the dressing was moist with clear fluid when the visiting nurse removed the bandage. The nurse returned two days later--the ninth postoperative day--and expressed concern when she re-examined the surgical site and observed that it was still moist. That same day the patient returned to the surgery clinic to be assessed.

During his evaluation in the clinic, fluid--now with a slight pink tinge--was noted at the surgical site on his back. Based on this finding--and an absence of any headache--the patient’s doctors diagnosed the fluid as a seroma. He was sent home.

The fluid, predominantly clear in appearance, persisted dripping onto the skin of his back. Two days later, on postoperative day 11, the patient and his wife (who was also a physician) independently contacted neurosurgeons whom they knew. The neurosurgeon contacted by the patient commented that although most patients with a cerebrospinal fluid leak presented with a headache, the absence of a headache did not exclude the possibility of a cerebrospinal fluid leak. The neurosurgeon contacted by the patient’s wife thought the diagnosis of a seroma was interesting and then adamantly stated that the patient had a cerebrospinal fluid leak. Both neurosurgeons insisted that the patient immediately return to the emergency center and not leave until the cerebrospinal fluid leak was diagnosed and treated.

The surgeon who evaluated the patient in the emergency center recommended the placement of several percutaneous sutures placed deeply into the subcutaneous tissue to tamponade the skin leak of cerebrospinal fluid. Eight sutures were placed (Figure 1B). There was no further leakage of cerebrospinal fluid onto the back skin.

However, as documented by the magnetic resonance imaging findings (Figures 2A, 3), the patient developed bilateral symptoms of sciatica from the compression of the spinal cord nerve roots by the cerebrospinal fluid in the surrounding soft tissue. A computed tomography-guided aspiration of some of the cerebrospinal fluid resulted in the resolution of the sciatica symptoms. Sequential magnetic resonance imaging--performed shortly after the aspiration of cerebrospinal fluid and three months later--both demonstrated a progressive decrease of the cerebrospinal fluid in the soft tissue.

## Discussion

Narrowing of the spinal canal results in lumbar spinal stenosis; this can result from degenerative changes in the facet spinal joints, the intervertebral spinal disks, and/or the ligamentum flavum. Indeed, the most common reason for spinal surgery in patients over age 65 years is lumbar spinal stenosis. In addition to decreased sensation and fatigue in the lower extremities, low back pain, and radiating pain in the legs (sciatica), additional symptoms of lumbar spinal stenosis may include impairment of urination and/or defecation and neurogenic--persistent or intermittent--claudication (with buttock and lower limb pain that is exacerbated when walking or standing for a long duration of time [3,4,8].

Sciatica is also referred to as lumbosacral radicular syndrome and radicular pain. Symptoms can be slow in onset or sudden in development; typically, the pain is sharp or aching or both and extends from the buttocks to the thighs and then radiates below the knee into the foot and toes. Low back pain may also be present; however, less than half of the patients with sciatica have associated lower extremity weakness [1,2,8]. 

Inflammation or compression of the fourth and fifth lumbar nerve roots and the first sacral nerve root can result in sciatica. Also, in addition to herniation of the disk and trauma, sciatica can result from stenosis of the foramina and soft tissue stenosis, such as a cyst, and extraspinal pathology (including the mass effect of a cerebral spinal fluid leak) or tumor. Sciatica is usually unilateral; however, central disk herniation, lumbar spinal stenosis, and spondylolisthesis can result in bilateral sciatica [1,2].

The diagnosis of lumbar spinal stenosis-related sciatica can be suspected based on symptoms and physical examination. For persistent or progressive sciatica, magnetic resonance imaging can be useful to define the etiology. Computed tomography and radiographs are usually not utilized. Although electrodiagnostic evaluation (such as needle electromyography and nerve conduction studies) can be used to determine whether sciatica is caused by radiculopathy instead of a musculoskeletal etiology, its role in the assessment of sciatica remains to be established [1,2,8].

Various conservative modalities have been used for the treatment of sciatica-associated lumbar spinal stenosis. These include exercise, medication, and physical therapy. In addition, there are several other non-operative treatments (Table 1) [3,4,8].

**Table 1:** Non-surgical treatment modalities for lumbar spinal stenosis ^a^The current neurosurgical literature suggests that bed rest is only recommended for severely debilitated patients. ^b^These include hyperextension exercise and isometric flexion exercise. ^c^These include anticonvulsants (such as gabapentin), antidepressants, anti-inflammatory drugs, corticosteroids, muscle relaxants, and prostaglandin E1 analogs.

Non-operative treatment
Bed rest^a^
Electrical stimulation
Epidural corticosteroid injection
Lifestyle modification
Lumbar exercise^b^
Massage
Medication^c^
Multidisciplinary rehabilitation
Orthosis usage
Physical therapy
Thermal therapy
Traction therapy

Management of lumbar spinal stenosis for patients with persistent symptoms, functional limitations, or both may require surgery. A recent meta-analysis showed that the optimal choice for interventional treatments for lumbar spinal stenosis included non-fusion methods (such as decompression and an interspinous process device). In addition, it noted that the use of an interspinous process device had both a low incidence of complications and a high rate of reoperation [5,8].

An epidural injection is a common non-surgical treatment for sciatica-associated lumbosacral spinal stenosis and radicular pain. In the early 1900s, the technique originated in Paris, and cocaine was injected into the sacral hiatus. Subsequently, in the 1950s, patients began to be treated with epidural corticosteroids [6].

Nerve edema--associated with spinal stenosis--results from the chemical irritation by inflamed nerve roots and the structural stimulation of physically compressed nerve tissue. Indeed, congestion of venous blood around the nerve roots causes local ischemia that subsequently elicits not only nerve edema but also inflammation from phospholipase and/or leukotriene B release. The mechanism of action whereby epidural corticosteroid injection diminishes nerve edema, and its associated inflammation, is by inhibiting the production and release of cytokines, reducing the migration of leukocytes, and stabilizing the membranes of cells [3].

An analysis was performed to assess whether epidural corticosteroid injections were effective for lumbosacral radicular pain. The study showed that, at short-term follow-up, the corticosteroid injection was slightly more effective than the placebo for leg pain; also, there were only minor adverse effects from the procedure. However, the long-term benefits of epidural corticosteroid injections have not been established. Therefore, epidural corticosteroid injection continues to be used for the management of patients whose daily life is interfered with by either acute radiating pain and/or neurological claudication [3,7,8].

The reported patient had an epidural corticosteroid injection on two occasions. After each of the injections, there was prompt relief of any sciatica-related pain. However, the lumbosacral stenosis-associated weakness did not resolve or improve following the injections; the lack of an efficacious response regarding the motor strength of his lower extremities prompted him to proceed with surgery.

An intraoperative tear of the dura occurred during the patient’s lumbar spinal surgery. This is commonly referred to as either an accidental dural tear or an incidental durotomy. The incidence of a dural tear during spinal surgery has been observed to range from 0.4 percent to 15.8 percent; a summarized incidence is approximately 5.8 percent [9,10,12].

Advancing age (although specific ages were not described), revision surgery, and lumbar stenosis have been recognized as significant risk factors for accidental dural tears. Diabetes mellitus was also weakly associated with incidental durotomy; however, neither the individual’s sex nor obesity were considered to be statistically significant [9]. Hence, although this was the patient’s first back surgery, he was 58 years old, and he had severe lumbar stenosis.

Similar to this patient, the dural tear may be noticed during the surgical procedure. Therefore, attempting to repair the tear, the iatrogenic defect in the dura was sutured during the operation. Subsequently, after the completion of surgery and for two additional days, he was placed in a slight Trendelenburg-like position with his feet higher than his head [13,20]. 

In other individuals, the cerebrospinal fluid leak from an intraoperative tear of the dura may not be recognized during the spinal surgery. However, the leak of cerebrospinal fluid into the surrounding space results in cranial hypovolemia and cranial hypotension with a corresponding reduction of intracranial pressure. Subsequently, a postural headache and/or other symptoms associated with the accidental dural tear with its related cerebrospinal fluid leak and spontaneous intracranial hypotension may occur (Table 2) [10-12]. 

**Table 2:** Symptoms associated with a tear of the dural tear and spontaneous intracranial hypotension ^a^Any movement of the head results in worsening of the headache. ^b^The headache occurs intermittently coinciding with intermittent spontaneous intracranial hypotension. ^c^This type of headache is also referred to as a postural headache; the headache worsens as the patient moves from either lying down or sitting to a standing position. ^d^In contrast to the orthostatic headache, the headache improves when the patient is in an upright position.

Symptom
Coma
Diaphoresis
Ears
Hyperacusis
Tinnitus
Eyes
Blurred vision
Diplopia
Gait
Ataxia
Staggering
Gastrointestinal
Nausea
Vomiting
Headache
Exertional^a^
Intermittent^b^
Orthostatic^c^
Paradoxical^d^
Movement
Chorea
Dystonia
Tremor
Neck
Pain
Stiffness
Photophobia
Quadriparesis
Vertigo

In addition to a postural headache and other symptoms associated with spontaneous intracranial hypotension, other clinical manifestations suggestive of a possible intraoperative dural tear may also occur following spinal surgery. These findings include clear fluid draining from the surgical wound, neurologic deficits, physical examination demonstrating a palpable fluid collection, symptoms experienced by the patient (such as axial pain and recurrence of preoperative symptoms), and wound drainage systems with unexpectedly high output. The observation of any of these findings should prompt the clinician--optimally the surgeon who operated--to immediately assess the patient and manage the complication in a timely and effective manner by not only entertaining the possibility of a cerebrospinal fluid leak but also considering prompt imaging, such as magnetic resonance imaging, for evaluation [20]. 

The reported patient only had persistent and continuous drainage from his surgical wound, which varied in presentation from being clear to slightly pink-tinged. When he returned to the surgery clinic for assessment, the physicians--based on the color of the dripping fluid--concluded that he had a seroma. Therefore, he was sent home and instructed to return to the clinic in a few weeks.

A seroma is a postoperative serous fluid collection; it is usually detectable on clinical examination. Surgical procedures in which there is the creation of an anatomical dead space may be associated with seroma formation. Therefore, seroma is commonly observed as a complication of plastic surgery procedures such as body contouring surgery for massive weight loss; however, seromas have also been noted postoperatively in lumbar spine surgery patients [17-19]. 

The diagnosis of seroma--in patients who had drains placed during surgery--may be suspected when a palpable or visible fluid wave is observed after the drains are removed. Radiologic examination--either utilizing ultrasonography or computed tomography--can also be used to establish the diagnosis; however, these modalities require ancillary equipment for evaluation and are expensive. Therefore, needle aspiration is not only a more cost-effective manner to consider for assessing the patient, but is also considered to be a conservative initial approach to the management of a seroma; however, aspiration of a subcutaneous collection of cerebrospinal fluid presenting as a draining duro-cutaneous fistula in the unsterile setting of a surgery clinic would not be recommended. If the seroma does not resolve after serial needle aspirations, treatment alternatives include inserting a seroma catheter for continuous drainage, injection of sclerosing agents, or surgical interventions such as marsupialization and packing the wound or returning to the operating room to excise the seroma cavity [17-19]. 

Hence, the patient’s cerebrospinal fluid leak was initially misinterpreted as a seroma. This occurred since the fluid dripping from his surgical wound was slightly tinged pink--instead of being completely clear--and he had not experienced a headache or any other signs and symptoms typically associated with a postoperative cerebrospinal fluid leak. Fortunately for the patient, neither aspiration of the fluid nor injection of a sclerosant solution was performed during his surgery clinic visit. Indeed, the color of the fluid and the absence of any other symptoms resulted in a delay in the diagnosis of the patient’s cerebrospinal fluid leak his doctors; it was not until he decided to return to the emergency center and insisted on a neuroimaging evaluation of his lower back, that the correct diagnosis of a cerebrospinal fluid leak--from the intraoperative dural tear--was established.

Serious complications can be associated with an accidental dural tear. Several of these potential consequences are summarized in Table 3 [10,12]. The patient in this report developed cerebrospinal fluid fistulas from the dura to the skin with a persistent and continuous leak of fluid.

**Table 3:** Complications associated with dural tears

Complication	Comment
Duro-cutaneous fistula	The formation of a fistula between the dura and the skin can subsequently result in either arachnoiditis, or epidural abscess, or meningitis.
Cerebrospinal fluid leak	A continuous leak predisposes to the development of pseudomeningocele formation and possible trapping of nerve roots with associated neurologic symptoms. The symptoms may include sciatica and cranial nerve palsies; for example, strabismus may occur as a result of a cranial nerve VI palsy.
Headache	A debilitating orthostatic headache often arises. The headache is postural; when the patient sits upright, it occurs, and when the patient is recumbent, it resolves.
Subcutaneous fluid collection	Proper wound healing may be prevented by fluid collection. This can result in breakdown of the wound, incision site infection, or both.

The patient’s cerebrospinal fluid leak was easily diagnosed after viewing the magnetic resonance imaging of his lower back. Indeed, several imaging techniques can be used to diagnose a cerebrospinal fluid leak following an accidental dural tear. These include magnetic resonance imaging (brain or spine), cisternography (radioisotope), and myelography (computed tomography or digital subtraction or dynamic computed tomography or magnetic resonance--T2-weighted) [10,11].

Other methods for diagnosing a cerebrospinal fluid leak include analysis of the fluid. The biochemical tests evaluate the fluid for the presence of either glucose oxidase, beta-2 transferrin, or beta-trace protein. The glucose oxidase test is inexpensive, rapid, and easy to perform; however, both the sensitivity and specificity of the test are low secondary to false positives (when there is bacterial contamination) and false negatives (in diabetic patients). Although the beta-trace protein test is highly sensitive, rapid (taking only 20 minutes to perform on a nephelometer), and inexpensive; yet, the diagnostic usefulness is limited not only by associated problems with assessing accurate cut-off values but also by increased serum and decreased cerebrospinal fluid beta-trace protein values are observed in a patient with renal insufficiency and bacterial meningitis [14,15].

Detecting beta-2 transferrin is the gold standard for confirming a cerebrospinal fluid leak; however, the gel electrophoresis technique is difficult and labor-intensive, expensive, and requires three to six hours to perform. Yet, a new platform for the detection of beta-2 transferrin has been developed that enables the rapid identification of cerebrospinal fluid leakage. None of these tests were needed to be performed to confirm the cerebrospinal fluid leak in the reported patient after the evaluation of his magnetic resonance imaging [14,16].

Direct suture repair is a common treatment approach for a cerebrospinal fluid leak after spine surgery; however, there is nearly a ten percent failure rate. Therefore, some surgeons will use adjuvant dural closure material such as polyethylene glycol as a hydrogel sealant. If the dural defect cannot be repaired directly, an augmented closure with either fat, muscle tissue, or fascial graft may be performed [11,13,20].

Management based on fluid flow mechanics can also be incorporated into the approach to treating a dural tear with accompanying cerebrospinal fluid leak. This involves decreasing the difference between the pressure of the subarachnoid fluid and the epidural fluid. Hence, the cerebrospinal fluid leak can be slowed by either reducing the subarachnoid fluid pressure or by increasing the epidural fluid pressure, or both [11,13,20].

Adjusting the position of the patient, using a subarachnoid catheter to shunt the cerebrospinal fluid, or inhibiting cerebrospinal fluid formation (by using acetazolamide) can reduce subarachnoid fluid pressure. For example, patients with a lumbar or thoracic cerebrospinal fluid leak should be kept in either Trendelenburg or prone position. The reported patient was placed on his back in the Trendelenburg position for at least two-and-a-half days after his surgery was completed [11,13,20].

An increase in epidural fluid pressure--accompanied by slowing of cerebrospinal fluid flow and facilitation of dural flap adherence--can result from a tight fascial closure. In addition, elimination of the dead space can result by incorporating a subfascial drain and discharging the excess cerebrospinal fluid. Another alternative to seal the cerebrospinal fluid leak and concurrently increase the epidural fluid pressure is an epidural blood patch [11,13,20]. 

The patient refused to return to the operating room for a surgical evacuation of the cerebrospinal fluid in an attempt at an operative repair of the torn dura. Therefore, the patient’s duro-cutaneous fistulas were successfully treated with tamponade sutures that compressed the affected areas. Subsequently, computed tomography-guided partial removal of the extra-dural collections of cerebrospinal fluid provided relief of the recurrent sciatica symptoms from the compression of his nerve roots from the fluid. Follow-up magnetic resonance imaging shortly after the procedure confirmed that some of the cerebrospinal fluid had been removed without the accumulation of new fluid; another magnetic resonance imaging, three months later, demonstrated less cerebrospinal fluid, indicating some spontaneous absorption of the residual fluid.

Four and a half years after surgery, the patient remains free of sciatica nerve symptoms. However, paresthesia affecting both his left distal foot and right middle three toes appeared on the evening of his surgery and continues to persist with altered proprioception of the affected areas. In addition, he did not regain the motor strength of his legs; it takes him at least 15 minutes to ambulate a mile.

**Figure 4:**
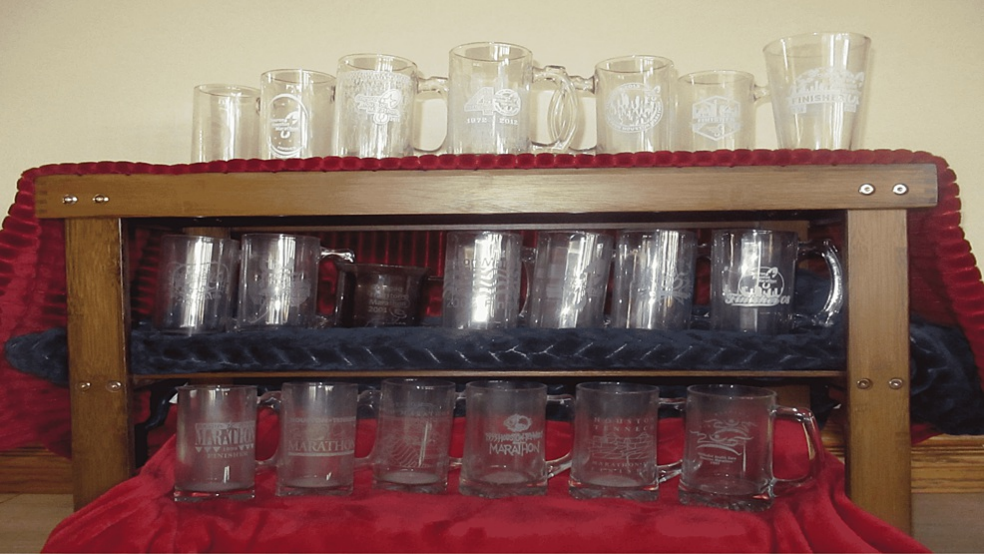
Finisher mugs from the 20 marathons completed in Houston, Texas A mug was awarded to the patient after he completed each January marathon in Houston, Texas, beginning in 1990 and ending with the final race in 2015. The 20 mugs were earned for finishing the following races: bottom row, left to right: Houston-Tenneco marathon (1990 to 1994) and Methodist Health Care Houston Marathon (1997); middle row, left to right: Methodist Health Care Houston Marathon (1998, 2000), Compaq Houston Marathon (2001, 2002), and Chevron Houston Marathon (2006 to 2008); top row, left to right: Chevron Houston Marathon (2009 to 2015).

**Figure 5:**
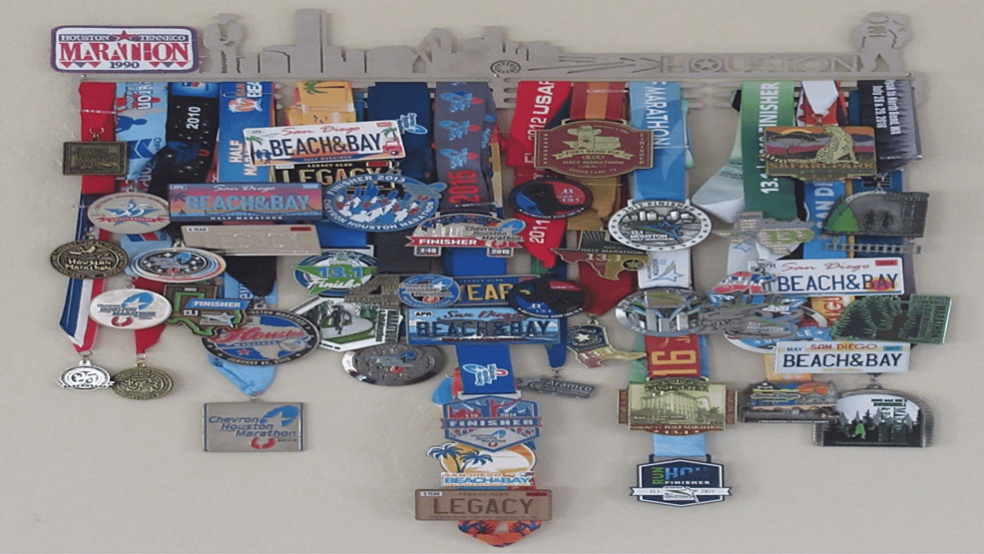
Finisher medals for marathons and half marathons A wall hanging shows the numerous medals received for completion of either marathons or half marathons by the patient. His first race was the Houston-Tenneco marathon in Houston, Texas, in January 1990, which he completed in three hours and 46 min. After his back surgery in 2017, he did not regain the motor strength in his lower extremities and still experiences paresthesia of his distal feet. However, he continues to participate in a few half marathons each year; indeed, the most recent race was his sixth consecutive Beach and Bay half marathon in San Diego, California, in April 2022, which he completed in three hours and 35 minutes.

Before his back surgery, the patient had completed 20 marathons in Houston, Texas (Figure 4). He realizes that he will probably never run another marathon. However, as long as he is capable, he is committed to continuing to participate in two or three half marathons each year (Figure 5).

## Conclusions

Lumbar spinal stenosis is a significant problem for ageing individuals. Non-operative treatments (such as exercise, medication, and physical therapy) may be effective for the associated lower back and leg symptoms. Epidural corticosteroid injections are also commonly used; although they often provide short-term pain relief, the long-term benefit has not been demonstrated. Spinal decompression surgery may be necessary if symptoms persist and/or functional limitations develop despite non-operative interventions. A potential complication of lumbar spinal stenosis surgery is a cerebrospinal fluid leak. A man (who is the first author of this paper) is described who experienced an intra-operative accidental dural tear during lumbar spinal stenosis surgery and subsequently developed a large cerebrospinal fluid leak that involved both the laminectomy bed and the subcutaneous tissue of his back that presented as duro-cutaneous fistulas without the headache which his doctors misinterpreted as a seroma. In summary, the absence of a headache does not exclude the possibility of a cerebrospinal fluid leak following lumbar spinal stenosis surgery. And although a seroma may occur following lumbar spinal stenosis back surgery, the presence of fluid leaking from the surgical site should not only prompt consideration of a surgery-associated cerebrospinal fluid leak but also additional evaluation--such as imaging studies--to confirm the diagnosis.
